# Recent Advances and Challenges in Controlling the Spatiotemporal Release of Combinatorial Anticancer Drugs from Nanoparticles

**DOI:** 10.3390/pharmaceutics12121156

**Published:** 2020-11-27

**Authors:** Moon Sup Yoon, Yu Jin Lee, Hee Ji Shin, Chun-Woong Park, Sang-Bae Han, Jae-Kyung Jung, Jin-Seok Kim, Dae Hwan Shin

**Affiliations:** 1 College of Pharmacy, Chungbuk National University, Cheongju 28160, Korea; dy5393@gmail.com (M.S.Y.); 5yujinli15@gmail.com (Y.J.L.); xxoodd1@gmail.com (H.J.S.); cwpark@chungbuk.ac.kr (C.-W.P.); shan@chungbuk.ac.kr (S.-B.H.); orgjkjung@chungbuk.ac.kr (J.-K.J.); 2 Drug Information Research Institute, College of Pharmacy, Sookmyung Women’s University, Seoul 04310, Korea

**Keywords:** nano-formulated combinatorial drug, ratiometric, sequential, spatiotemporal, controlled release

## Abstract

To overcome cancer, various chemotherapeutic studies are in progress; among these, studies on nano-formulated combinatorial drugs (NFCDs) are being actively pursued. NFCDs function via a fusion technology that includes a drug delivery system using nanoparticles as a carrier and a combinatorial drug therapy using two or more drugs. It not only includes the advantages of these two technologies, such as ensuring stability of drugs, selectively transporting drugs to cancer cells, and synergistic effects of two or more drugs, but also has the additional benefit of enabling the spatiotemporal and controlled release of drugs. This spatial and temporal drug release from NFCDs depends on the application of nanotechnology and the composition of the combination drug. In this review, recent advances and challenges in the control of spatiotemporal drug release from NFCDs are provided. To this end, the types of combinatorial drug release for various NFCDs are classified in terms of time and space, and the detailed programming techniques used for this are described. In addition, the advantages of the time and space differences in drug release in terms of anticancer efficacy are introduced in depth.

## 1. Introduction

Many studies are being conducted to overcome cancer, a major health problem for humans in modern society. Chemotherapy has been in the spotlight as the main approach for anticancer research, but there are several limitations to this approach [1]. As chemotherapy usually uses a single anticancer drug that targets only one signaling mechanism, many problems such as drug resistance, side effects on healthy tissues, and poor pharmacokinetic profiles are encountered [2,3,4]. Therefore, combination therapy using two or more different drugs has been considered as a solution for anticancer therapy [5]. However, combination therapy also has its disadvantages. It is difficult to control the pharmacokinetics and pharmacodynamics due to a time difference in drug administration, in which individual drugs are administered in combination, and the possibility of cross-resistance induced by the administration of several drugs [6,7]. Owing to its high level of adaptability, cancer is difficult to treat owing to multiple drug resistance (MDR) that results in simultaneous resistance to multiple drugs with various chemical structures and the associated mechanisms of action [8]. To solve these problems, recent research has focused on methods using nanoparticles as nanocarriers in anticancer therapy [9,10].

Nanoparticles have been widely used as vehicles for cancer treatment because of their several advantages, and various nanocarriers such as liposomes, polymeric micelles, silica nanoparticles, chitosan nanoparticles, and protein nanoparticles are being developed [11,12,13,14,15]. The encapsulation of drugs using nanocarriers can help solubilize various poorly water-soluble drugs using nanocarriers with amphiphilic properties [16]. In addition, it is possible to protect drugs through nano-encapsulation or via nanoparticle uptake through endocytosis [17]. A key advantage of using nanoparticles in chemotherapy is the ability to differentiate cancer cells from normal cells and selectively remove cancer cells [18]. Nano-formulated drugs can avoid rapid removal from the body through an enhanced permeability and retention effect (EPR), allowing passive tumor accumulation into tumors, as well as active tumor accumulation by adding ligands for targeting cancer cells [19].

Nano-formulated combinatorial drugs (NFCDs), which can be co-delivered with these nanoparticles, have several unique advantages, such as improving synergistic treatment efficacy, drug resistance management, and the ability to temporarily control drug release [20]. NFCD treatments are considered to have high potential to solve problems such as drug toxicity and dose control, as they can simultaneously utilize the advantages of existing nanoparticles and combination therapy [8,21,22]. NFCDs in various formulations can be prepared, depending on the type of nanoparticles to be used and the drug to be combined. Due to these advantages, studies on NFCD are being actively conducted, and some studies have already reached the clinical trial stage [23,24,25,26,27].

**Figure 1:**
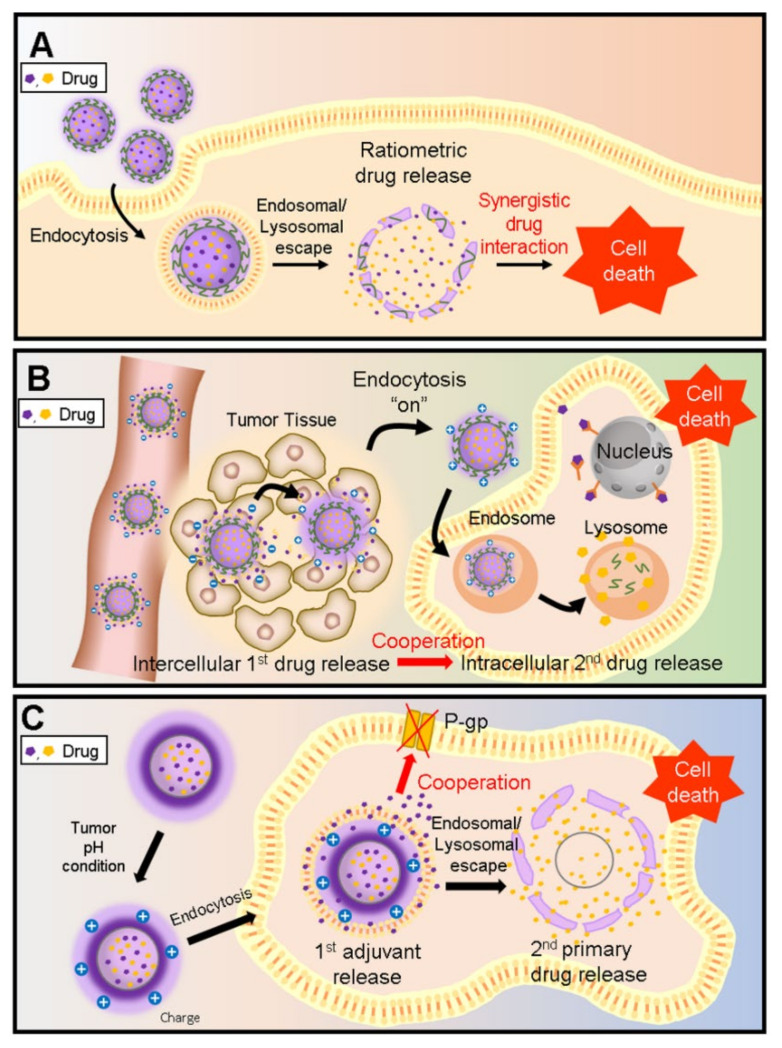
Schematic illustration of various spatiotemporal types of combinatorial anticancer drug release. (**A**) Ratiometric drug delivery and simultaneous release for synergistic drug interaction. (**B**) Sequential drug release to achieve both intercellular and intracellular drug action. (**C**) Intracellular sequential release of adjuvant and primary drug for enhanced drug efficacy.

We focused on the controlled release of NFCD and confirmed previous studies. NFCDs can be released at the same time or sequentially at intervals of time. In addition, drugs can be released into different spaces. As the anticancer efficacy of NFCDs can vary greatly depending on the drug release pattern from nanoparticles, it is necessary to carefully consider the drug release system according to time and space. Therefore, in this review paper, we classify the types of controlled release of NFCD as ratiometric drug delivery that is simultaneously released over time, sequential drug delivery that is released within cells in order, and sequential drug delivery with intercellular sequential delivery followed by intracellular sequential delivery. We also introduce the advanced technologies of controlled release for this and review the benefits of each controlled drug release pattern and the prospects of these technologies (Figure 1) (Table 1).

## 2. Ratiometric Drug Delivery

**Figure 2:**
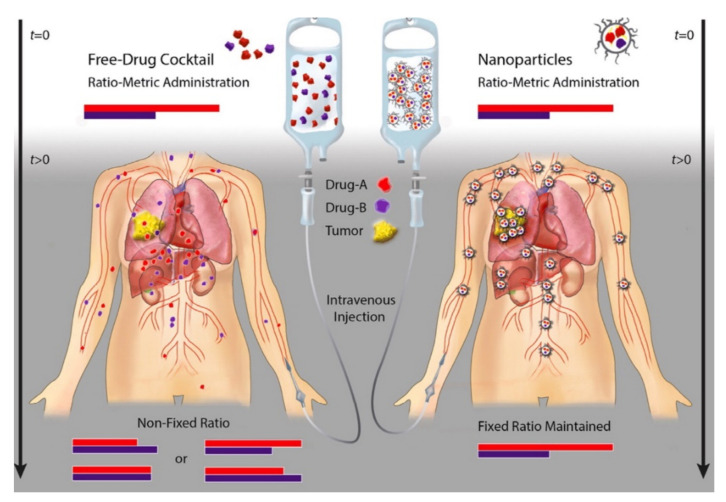
Ratiometric drug delivery of combinatorial drugs using nanoparticles is more advantageous in terms of pharmacokinetics and biodistribution of drug combinations compared to free combinatorial drugs. Reproduced with permission from [40], Journal of Controlled Release, 2016.

By inhibiting cancer via different mechanisms through multi anticancer agents, resistance to anticancer agents can be reduced to a higher degree than when using a single anticancer agent; moreover, a synergistic effect can occur, leading to higher efficiency. For specific combinations to achieve a synergistic anticancer effect, the drugs must be delivered to cancer cells at a fixed constant rate. However, this is difficult owing to the different pharmacokinetic properties of drugs [41]. In addition, the toxicity of drugs to normal cells can cause problems. These problems can be solved using nanoparticles as a carrier for combination drugs. Ratiometric drug release is a drug delivery system that simultaneously releases drugs that are encapsulated in a nanocarrier, and this method can integrate the pharmacokinetics of different drugs (Figure 2) [42]. In the case of ratiometric drug delivery, examples were divided according to how combinatorial drugs were simultaneously released intracellularly, and the techniques used and drug efficacy were discussed.

### 2.1. Release of Co-Loaded Drugs through pH Control

Penetration of drugs into tumor tissue is considerably difficult owing to the abnormal extracellular matrix and high cancer cell density [43,44]. Therefore, for better efficacy, anticancer drugs should act more selectively on cancer cells than on normal cells. Tumor tissue has a relatively low pH than normal cells; therefore, anticancer agents should promote drug release under acidic pH conditions.

**Figure 3:**
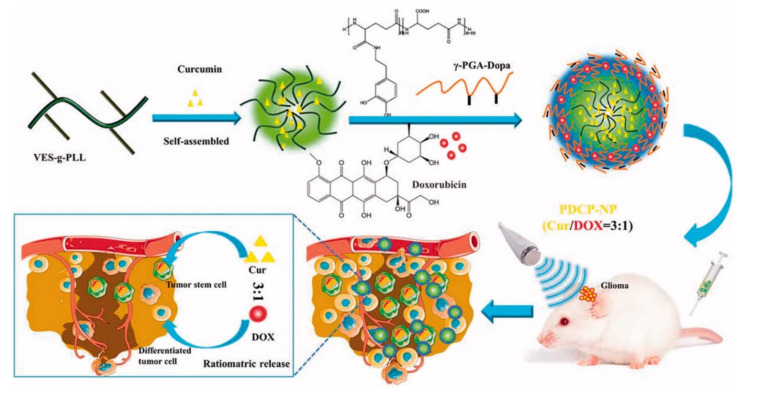
Schematic diagram of pH-sensitive core–shell nanoparticles for ratiometric drug release. The curcumin/doxorubicin co-loaded on the pH-sensitive core–shell nanoparticles is released at a constant ratio in cancer cells. Reproduced with permission from [28], Drug delivery, 2018.

To effectively penetrate tumor tissues, Xu et al. prepared cationic nanoparticles of VES-g-ε-PLL (Cur-NPs) encapsulating the well-known natural anticancer agent hydrophobic curcumin (Cur) in vitamin E succinate-grafted-ε-polylysine (VES-g-ε-PLL) [28,45]. Then, pH-sensitive core–shell nanoparticles (PDCP-NPs) were formed using doxorubicin (DOX) hydrochlorate and Cur-NPs in dopamine-modified-poly-γ-glutamic acid polymer (γ-PGA-Dopa) (Figure 3). In these nanoparticles, γ-PGA provides a drug-loading site for most primary chemotherapeutic drugs via carboxyl–metal ion coordination or electrostatic interactions. In general, these nanoparticles differ from other nanoparticles combining two drugs in a polymer because DOX is encapsulated in the outer shell. γ-PGA has high biocompatibility and biodegradability; thus, it is nontoxic to the human body and contributes to the stability of PDCP-NPs in vivo and in vitro. In addition, γ-PGA improves drug delivery efficiency by contributing to the intracellular absorption of cancer cells. The side carboxyl groups of γ-PGA coated on PDCP-NPs are protonated in acidic conditions to promote the rapid release of DOX. Free amino groups of exposed Cur-NP are also protonated to increase the release rate of Cur from PDCP. Through this process, drugs with different physical properties can be released proportionally. Cur and DOX encapsulated at a ratio of 3:1 in PDCP-NPs were released at a ratio close to 3:1 in cancer cells, and they subsequently inhibited the rapid proliferation of cancer cells and caused apoptosis. In vivo, the PDCP-NP treatment group showed stronger antitumor effects than the single-drug-loaded nanoparticle treatment group. Thus, simultaneous delivery of Cur and DOX showed better treatment efficiency than administration of single-drug-loaded nanoparticles. In addition, tumor volume increased over time in the brain of glioma rats treated with CUR/DOX complex liquid and bilayer pH-sensitive DOX nanoparticles, whereas tumor growth inhibition was observed in mice with treated with PDCP-NPs. Therefore, it was confirmed that the survival rate of mice after PDCP-NP treatment was prolonged compared that of mice after control treatment [28].

Nanoparticles can accumulate in tumors through the EPR effect, but inefficient intracellular release results in inefficient treatment [46,47,48,49]. To solve this problem, Guo et al. studied positively charged polymer nanoparticles to improve drug bioavailability through strong adsorption of negatively charged cell membranes and cationic polymer nanoparticles [29]. The surface of cationic nanoparticles is usually decorated with amino-rich functional groups. One example is ε-poly-l-lysine (EPLYS), a naturally biodegradable homopoly(amino acid), which demonstrated no cytotoxicity with the resultant nanoparticles [29].

Thus, Guo et al. fabricated novel dual drug-loading polymeric nanoparticles using polyethylene glycol (PEG) and EPLYS that physically encapsulated lapatinib (LAP) and DOX (DMMA-P-DOX/LAP nanoparticles) [50,51,52,53,54,55,56,57]. In these polymer–drug conjugates, an acid-cleavable linker was inserted between the drug molecule and the polymer, accelerating the decomposition of the conjugate under intracellular pH conditions to accurately deliver and release drugs [58,59,60,61,62]. Therefore, DOX was conjugated to the hydrophilic PEG-EPLYS backbone through acid-labile imine bonds, and LAP was physically encapsulated into the nanoparticles; thus, after the cleavage of imine bond, the remaining hydrophobic chain was rendered insufficient, leading to rapid decomposition of nanoparticles. Through these processes, DOX and LAP were simultaneously released. The DMMA-P-DOX/LAP nanoparticle showed that, following intravenous injection, nanoparticles accumulated in the tumor tissue through the EPR effect, and the surface charge reversed from negative to positive, enhancing tumor cell internalization [29,63]. As a result, the low pH of the cells caused the cleavage of residual amino groups, thus rapidly breaking down nanoparticles. Therefore, DOX and LAP were simultaneously released accurately into the cytoplasm, effectively inhibiting cell proliferation. As a result of confirming the antitumor effect in vivo, the tumor was more suppressed in the group treated with DOX and LAP nanoparticles compared to the group treated with only free DOX, free LAP, and DOX nanoparticles. In addition, it was confirmed that tumor volume in the DMMA-P-DOX/LAP nanoparticle group decreased more rapidly than that in the DMMA-P-DOX nanoparticle group, and the tumor was completely removed after chemotherapy [29].

Nanoparticles are characterized by stimulus responsiveness for effective drug release at the target site, releasing drugs with environmental changes. Among various stimuli, the pH of the endosome/lysosome (pH 5.0) in cancer cells is relatively lower than that of the extracellular environment; therefore, pH responsiveness is most often used for ensuring drug release from nanoparticles [47,64,65].

### 2.2. Release of Co-Loaded Drugs through Polymeric Degradation

**Figure 4:**
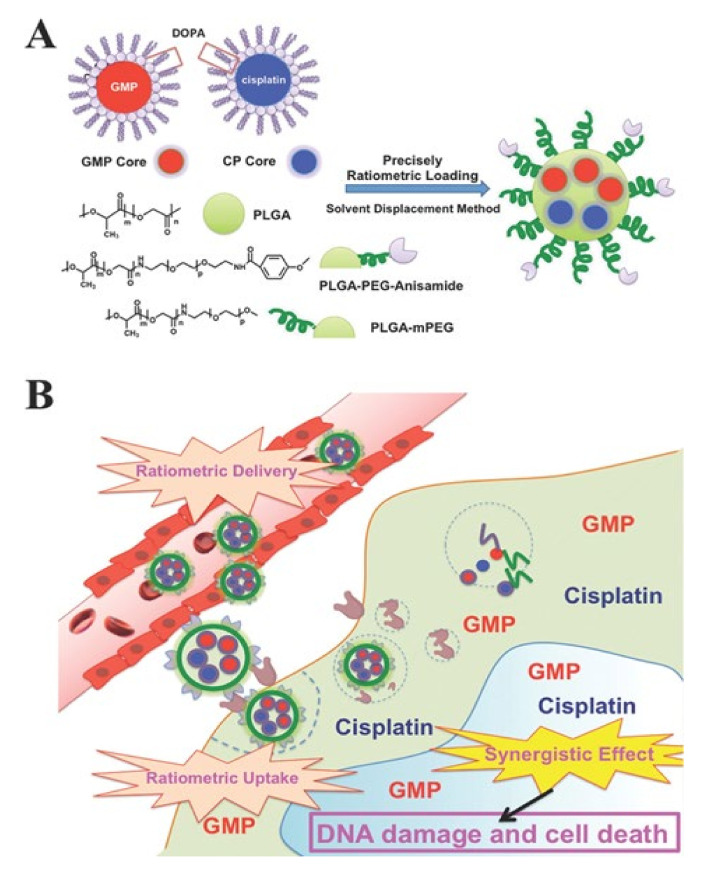
(**A**) Schematic diagram of PLGA-PEG-Anisamide NP (PLGA NP) including a dioleoyl phosphatidic acid (DOPA)-coated cisplatin core (CP core) and DOPA-coated gemcitabine monophosphate core (GMP core) through solvent substitution method. (**B**) Ratiometric drug delivery of CP core and GMP core co-loaded PLGA nanoparticles (combination NP) into cancer cells. Reproduced with permission from [30], Advanced Functional Materials, 2014.

It is considerably difficult to formulate nanoparticles using drugs with different physicochemical properties [5]. For example, cisplatin is characterized by limited solubility in both water and oil, and gemcitabine monophosphate (GMP) is a hydrophilic drug [66,67]. Cisplatin and GMP have different physicochemical properties and have limitations for loading in nanoparticles. To solve this problem, Miao et al. formulated nanoparticles after wrapping cisplatin and GMP with different characteristics using dioleoyl phosphatidic acid (DOPA) (Figure 4) [30]. Therefore, poly(lactic-*co*-glycolic acid) (PLGA) nanoparticles were formed using a DOPA-coated cisplatin core (CP core), DOPA-coated GMP core (GMP core), and PLGA. To further improve the internalization of PLGA nanoparticles into cancer cells, a ligand that acts with a receptor overexpressed on the surface of cancer cells was introduced into PLGA nanoparticles. The prerequisite for control of delivery in this way is to incorporate the physicochemical properties of the dual drugs by taking advantage of the similarities of the surface and size of the core. The advantage of this method is that it avoids functional indirection between individual molecules, allowing for precise ratio loading and delivery. In addition, the optimal combination drug ratio was more effective than single nanoparticles loaded with GMP and cisplatin separately, and it showed remarkable anticancer efficacy [2,68].

It was confirmed that the IC_50_ value of PLGA NP loaded into CP cores and GMP cores (combo NP) was smaller than the GMP nanoparticle and cisplatin nanoparticle. In addition to in vivo antitumor efficacy for tumor transplant models, combo free administration showed that the weight of the tumor was lower than that of the tumor when free cisplatin and free GMP were injected. On the other hand, the IC_50_ value of combo NP was larger than that of combo free, but there was no significant difference. The tumor weight was lower when a separate NP was injected than that when cisplatin NP and GMP NP were administered, and the tumor weight when combo NP was administered was the lowest. These results show that combination NPs containing cisplatin and GMP exhibited improved anticancer effects compared with a single drug.

In another study, rapamycin (RAPA), an mTOR inhibitor, was combined with cisplatin. It was shown that co-delivery of RAPA with cisplatin could significantly promote the efficacy of RAPA through microenvironment regulation [69,70]. However, encapsulating these drugs in PLGA NPs was inefficient owing to the incompatibility between the two drugs and the polymer matrix. Here, as in the previous example, the nano precipitate (cores) of the drug was coated with DOPA to make cisplatin hydrophobic [66,71,72]. Guo et al. attempted to co-encapsulate DOPA-coated cisplatin and RAPA in PLGA NPs using a solvent displacement method to improve the encapsulation and loading efficiency of the hydrophobic drugs [31]. The combination NPs showed sustained release of both cisplatin and RAPA, with similar release rates. This release rate suggests that the explosive release of cisplatin from PLGA NPs was prevented by the hydrophobic DOPA coating. In addition, the IC_50_ of combined drug was lower than that of the single drugs, and the IC_50_ of the PLGA-NP-encapsulated drug was lower than that of the free drug, showing better anticancer effect. In addition, in the in vivo experiment, it was confirmed that the tumor size decreased when (RAPA + cisplatin) NP was administered compared to when cisplatin NPs, RAPA NPs, and RAPA NPs + cisplatin NPs were administered [31].

Therefore, encapsulation of a hydrophilic phosphorylated drug generated via lipid coating of the surface layer of the calcium phosphate core using DOPA is an efficient method for achieving ratiometric drug delivery.

### 2.3. The Release of Co-Loaded Drugs through Enzymatic Degradation

Drug delivery systems that use polymer–drug conjugates not only have advantages such as reduction of drug toxicity, tumor accumulation through the EPR effect, and improvement of bioavailability, but they can also control the molar ratio of different drugs more elaborately than the drug encapsulation method using nanoparticles [73,74,75,76,77]. Drug–polymer conjugates have succeeded in maintaining the ratio between several different drugs, but the ratiometric release of different drugs from a carrier is still a difficult task due to the variability of drug and polymer interaction and the steric hindrance of drugs [20,78,79,80,81].

To compensate for this problem, Luo et al. studied a novel method of loading double drugs into a macromolecular carrier at different molar ratios of DOX and mitomycin C (MMC), which are widely known anticancer agents; however, they have serious side effects when administered as free drugs [32,76,82]. As a drug carrier, xyloglucan (XG), a natural and nontoxic polysaccharide was used, and tripeptide Gly-Leu-Gly which is degraded by lysosomal enzymes was used as a linker capable of attaching DOX and MMC to the XG [83,84,85]. XG-MMC/DOX was formulated using the anticancer drugs DOX and MMC, the carrier XG, and the linker tripeptide Gly-Leu-Gly that attaches the drug and the carrier. XG-MMC/DOX accumulates in the tumor via the EPR effect and reaches the lysosomal compartment of cancer cells, whereas the linker is degraded by the lysosomal enzymes, leading to drug release from the XG, thereby enabling efficient ratiometric drug release in cancer cells [86]. XG-MMC/DOX showed a superior anticancer effect in in vitro and in vivo cytotoxicity studies compared to cocktail mixtures of anticancer drugs such as XG-MMC and XG-DOX. Therefore, a polymer–drug conjugate complex, which uses a linker such as tripeptide Gly-Leu-Gly, is considered to have sufficient advantages for ratiometric drug delivery for anticancer therapy.

Ratiometric drug release is an important release technology that increases the efficacy of drugs by controlling the pharmacokinetics of two different drugs, delivering drugs better to the target than cocktail therapy. However, more advanced strategies are needed that further consider the MDR.

## 3. Sequential Drug Release

Although the co-administration of multiple drugs is a major strategy to overcome drug resistance, it might limit the synergistic effects of drugs in a heterogeneous tumor environment [81,87,88]. As the research on NFCDs continued, it was confirmed through advanced studies that not only were NFCDs released in a ratiometric manner in cancer cells but also sequentially (time or space differences). As drugs show differences in solubility or cancer-inhibitory mechanisms, there are cases where sequential release of drugs from NFCD complexes is advantageous. In addition, sequential release can be spatiotemporal, in which drugs are released only in the predetermined order of release within cancer cells. Therefore, we divided sequential drug release into intracellular sequential drug release, in which drugs are released only inside cancer cells in a certain release sequence, and spatiotemporal drug release, in which drugs are released site-specifically outside and inside cancer cells.

### 3.1. Intracellular Sequential Drug Release

The sequential release of NFCDs in cells is a temporal concept of drug release. The characteristic of this technology is that, unlike in conventional ratiometric release, the order in which drugs are released affects their anticancer efficacy. For example, in the delivery of a P-glycoprotein (P-gp) inhibitor and an anticancer drug, rather than releasing the two drugs ratiometrically into the cell at the same time, the P-gp inhibitor is released first to inhibit P-gp, followed by the release of the anticancer agent to more efficiently overcome MDR [89]. In addition, sequential release of drugs can lead to improved safety and reduced toxicity. Therefore, there is a need for nanoparticle formulations that can sequentially release several drugs at the target site. We divided the cases into the mechanism via which two drugs directly affect cancer cells through sequential release and the mechanism via which one drug amplifies the effect of other drug.

#### 3.1.1. Sequential Drug Release of Co-Loaded Drugs That Directly Affect Cancer Cells

**Figure 5:**
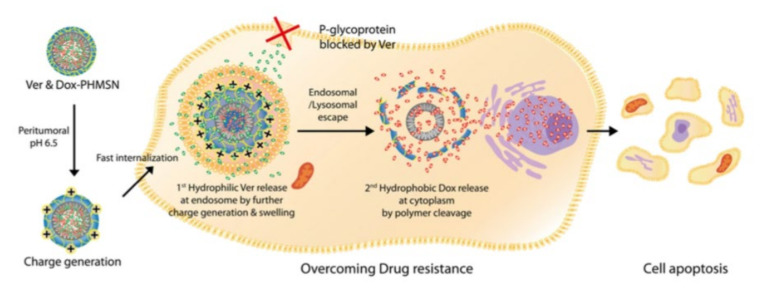
Sequential release of hydrophobic, hydrophilic drugs from polymer gatekeeper hollow mesoporous silica nanoparticles (PHMSNs). PHMSNs become positively charged in the pH condition of the tumor and are rapidly internalized into cells. Then, by the swelling of the polymer gatekeeper, the hydrophilic drug verepamil∙HCl is initially released to inhibit P-glycoprotein, and the hydrophobic anticancer drug doxorubicin is later released causing cell apoptosis. Reproduced with permission from [33], Advanced Functional Materials, 2017.

The research below describes an example of using hollow mesoporous silica nanoparticles (HMSNs) as part of a combination drug carrier (Figure 5). Hydrophilic anticancer agents can be loaded into HMSNs, and hydrophobic anticancer agents can be loaded through physical adsorption on the mesoporous surface of HMSNs, compared to conventional NFCDs that are loaded with poorly soluble drugs [90,91,92,93]. Pristine mesoporous silica nanoparticles (MSNs) have the disadvantage that the drug can be released before reaching a specific target [94]. Therefore, in order to overcome this drawback, polymer-coated HMSNs (PHMSNs) were prepared by coating a positively charged PEG-PDS-DPA copolymer on negatively charged HMSNs through electrostatic interactions. PHMSNs have stable colloidal properties, and selective drug delivery for specific targets (cancer cells) is possible [95]. It was proven through a pH-dependent cell uptake assay that co-drug-loaded PHMSNs do not release drugs under the pH conditions of normal tissue or blood (7.4), but exhibit the ability to release pH-reactive drugs under the pH conditions of tumors (5.5). In addition, after PHMSNs are internalized into cancer cells, the swelling of the polymer gatekeeper induces the release of the hydrophilic drug in the acidic environment of the endosome, and the hydrophilic drug is released first. In the cytoplasm, the polymer gatekeeper is cleaved by glutathione to release a hydrophobic drug, and it has been demonstrated that a controlled sequential release is possible in the cell.

Such intracellular sequential drug release of co-drug-loaded PHMSNs can be a solution to MDR. Multidrug efflux pumps, such as P-gp, reduce the drug concentration in the cytoplasm through the plasma membrane to reduce the anticancer effect of the drug [96]. Hydrophilic verapamil∙HCl, a calcium channel-blocking agent, is an inhibitor of P-gp and increases the accumulation of anticancer drugs in cancer cells by preventing anticancer drugs from escaping to the outside of the cell through the P-gp [97]. On conducting cytotoxicity assays of hydrophilic verapamil∙HCl, hydrophobic DOX co-loaded PHSMNs, and free DOX in P-gp overexpressed breast cancer cell lines, it was confirmed that the former has stronger cytotoxic effects. Therefore, this strategy, wherein verapamil∙HCl, an inhibitor of P-gp, is initially released, controls P-gp, and then sequentially releases anticancer drugs, is thought to be an effective option to treat cancer.

Janus nanoparticles (JNPs) can also be used to sequentially release hydrophilic and hydrophobic anticancer agents which can directly affect cancer cells. JNPs can have two or more different physical properties at the same time owing to their heterogenous structure and functionalities, which allow both hydrophilic and hydrophobic drugs to be loaded into the differing components of the structure and to be released via different stimuli [98,99,100,101,102]. DOX and docetaxel (DOC), the most well known anticancer drugs with different cancer cell-inhibitory mechanisms, showed a synergistic therapeutic effect with a combination index lower than 1 when used as a cocktail therapy; nevertheless, owing to their different solubility, the two drugs were difficult to be incorporated into nanoparticles, and their sequential release in cancer cells was even more difficult [103,104,105].

To prepare JNPs to co-load and sequentially release DOX and DOC, Zhang et al. first prepared Ag nanocube/poly(acrylic acid) (AgNC/PAA) by mixing AgNC and PAA. AgNC/Fe(OH)_3_-PAA was prepared by growing Fe(OH)_3_ in the PAA part of AgNC/PAA to ensure loading of hydrophilic DOX and release of DOX in response to pH stimulation [34]. Then, AgNC was etched with HAuCl_4_ to form AuNC through a galvanic exchange reaction to prepare AuNC/Fe(OH)_3_-PAA JNPs. Lastly, PCL-AuNC/Fe(OH)_3_-PAA JNPs were synthesized by modifying AuNC with PCL-SH to obtain the ability to load hydrophobic DOC, the photothermal effect, and the release properties allowing DOC release in response to near-infrared (NIR) stimulation [106].

DOC- and DOX-loaded PCL-AuNC/Fe(OH)_3_-PAA JNPs (DDPs) are taken up into the cytoplasm through lysosomes or endosomes, via the EPR effect. Hydrophilic DOX, loaded in the Fe(OH)_3_-PAA part of JNPs, is first released in response to low pH of the cytoplasm (pH 5.5). Next, upon NIR laser irradiation, the hydrophobic DOX loaded in the PCL-AuNC part of JNPs is released in response to NIR stimulation. NIR laser irradiation not only triggers DOC release but also causes an additional photothermal effect, resulting in a higher chemotherapy effect in cancer cells [107,108,109]. In H-22 liver cancer model mice, the anticancer efficacy of NIR laser-irradiated DDPs was superior to that of general DDPs, single free drugs, and free drug combinations. In addition, the DOC/JNP and DOX/JNP cocktail showed inferior drug efficacy to the combination of free drugs owing to unintended cell-cycle arrest, which was attributed to inappropriate sequential drug release and decreased response to subsequent drugs. Therefore, the photothermal effect induced by NIR laser irradiation was confirmed in DDPs, and it is important that DOC and DOX were released sequentially.

#### 3.1.2. Sequential Drug Release Where One of the Co-Loaded Drugs Amplifies the Effect of the Other Drug

Reactive oxygen is an unstable molecule. When reactive oxygen species accumulate in a cell, DNA, RNA, and proteins are damaged, and apoptosis can occur [110]. Using the specific role of these ROS, we introduce an NFCD delivery system that acts specifically on cancer cells. Stimulus-responsive drug delivery systems are technologies that target cancer cells by releasing stimulus-responsive drugs accumulated in cancer cells by glutathione (GSH), pH, and ROS [111,112,113]. In cancer cells and normal cells, when GSH-based and pH-based drug release systems are used, the selectivity is not high; however, ROS such as hydrogen peroxides (H_2_O_2_) and hydroxyl radicals (OH^•^) can have high selectivity because cancer cells have more than 10-fold the levels of normal cells [114,115,116,117,118]. However, cancer cells have heterogeneity and, thus, it might be difficult to fully realize the ROS-responsive release effect [119]. Therefore, to increase the selectivity for cancer cells, a cascade amplification strategy was used to increase the ROS concentrations to a greater extent [120].

**Figure 6:**
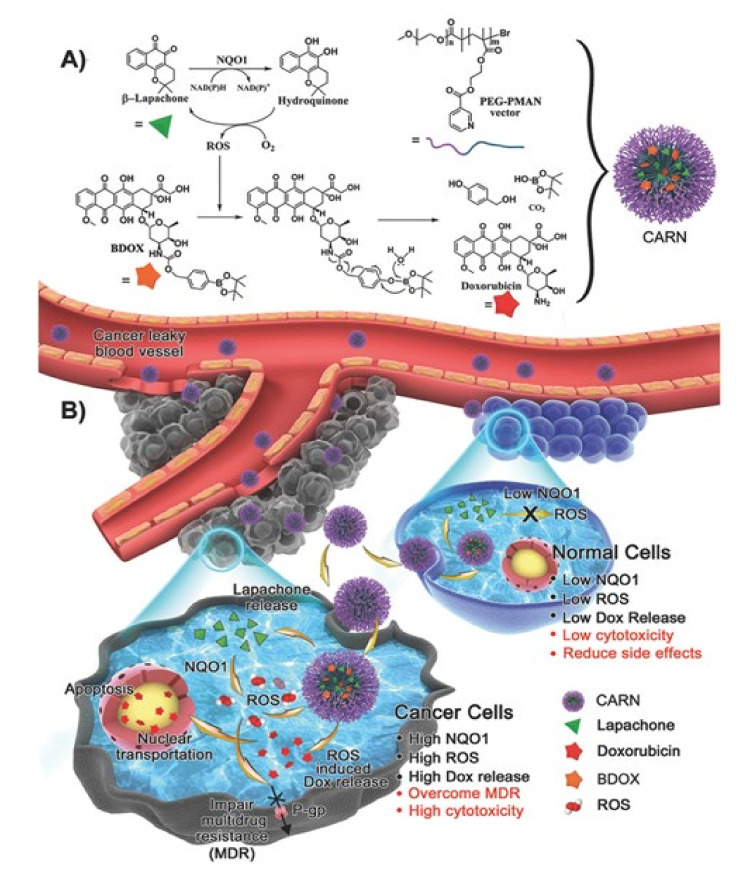
Cascade amplification release nanoparticle (CARN) formation process and its sequential release system. (**A**) Structure of BDOX, *β*-lapachone, and PEG-PMAN constituting CARN. (**B**) CARNs, which move through blood vessels, are accumulated in cancer cells. When β-lapachone is initially released from the nanoparticles, it amplifies reactive oxygen species (ROS). The amplified ROS blocks P-glycoprotein (P-gp), preventing DOX from escaping out of the cell. It also changes BDOX to DOX, causing apoptosis. Reproduced with permission from [35], Advanced Materials, 2017.

Drugs that amplify ROS include d-amino acid oxidase, vitamin C, *β*-lapachone, cinnamaldehyde, etc. [121,122,123,124]. Ye et al. employed a technique using cascade amplification release nanoparticle (CARN), which is formed by encapsulating the ROS-responsive DOX prodrug (BDOX) and *β*-lapachone together in poly(ethylene glycol)-poly[[2-(methylacryloyl)ethylnicotinate] (PEG-PMAN) [35]. *β*-Lapachone has a very low encapsulation efficiency for commonly used polymers such as poly(ethylene glycol)-block-poly(lactic acid) (PEG-*b*-PLA). Therefore, PEG-PMAN was prepared using atom transfer radical polymerization to increase the encapsulation efficiency (Figure 6). When CARNs are injected intravenously, they travel through the blood vessels and accumulate in cancer cells due to the EPR effect of the cancer cell membrane. After that, *β*-lapachone, released first from the nanoparticles, amplifies ROS using the nicotinamide adenine dinucleotide (phosphate) (NAD(P)H):quinone oxidoreductase-1 (NQO1) enzyme, ultimately promoting DOX release from BDOX [125]. Unlike DOX, which is released in a pH-dependent manner, BDOX can suppress unwanted drug release as the release is not affected by changes in pH [35]. Since NQO1 is overexpressed in cancer cells compared to levels in normal cells, *β*-lapachone can function effectively in cancer cells [126]. In other words, CARNs that cannot generate sufficient ROS in normal cells with low NQO1 expression show low cytotoxicity toward normal cells. ROS amplified by *β*-lapachone blocks the function of P-gp, thereby preventing the drug from being released from the cells and consequently lowering MDR [127]. In addition, ROS moves DOX into the nucleus and DOX affects DNA and topoisomerase II, causing apoptosis [128,129,130,131,132,133]. DOX causes necrosis of cardiomyocytes due to its side effect that causes cardiac toxicity [134]. However, according to the results of a histological study, CARNs administered to mice did not cause DOX-induced cardiac toxicity. In addition, when phosphate-buffered saline (PBS), DOX, and CARN were administered to mice, there was no significant difference in body weight between PBS and CARN. In contrast, DOX showed significant body weight reduction. Therefore, it could be confirmed that CARN does not show toxicity.

When creating an effective drug delivery system, it is better to find a material with good biodegradability and biocompatibility [135,136]. Glucan, one of the materials with excellent biocompatibility and water solubility, is a polysaccharide that many researchers are exploring [137,138]. In particular, because acetylated glucan is sensitive to pH, it changes from hydrophobic to hydrophilic in a low-acidic environment. In addition, polymers such as poly(glycolic acid) or poly(lactic acid) are used as materials to increase biodegradability [139]. However, the decomposition products of these polymers under acidic conditions can cause side effects such as allergic reactions and inflammation [140,141,142,143]. In contrast, polyamino acid chains are characterized by remarkable biocompatibility, perfect biodegradability, and low toxicity [144,145]. Therefore, Luan et al. formed nanoparticle(P-NM-Lapa) with polyaspartic acid-acetylated maltoheptaose (PAsp-AcMH) composed of (AcMH) and (PAsp), mPEG-AcMH composed of AcMH and nontoxic PEG, and *β*-lapachone and positively charged nitrogen mustard (NM) prodrug [36]. P-NM-Lapa decomposes AcMH in an acidic environment, releasing *β*-lapachone and NM prodrugs. Although NM is an alkylating agent with anticancer effects, it has a short half-life in blood and can cause mutations on its own [146,147,148,149,150]. It also responds well to the DNA of normal cells and tumor cells, making it difficult to effectively target tumor cells [151]. However, P-NM-Lapa does not cause toxicity in normal tissues. In histological study, when administered to mice, vacuolization occurred in the tumor, but there was no significant difference from PBS in the liver, heart, and kidney. Therefore, this means that P-NM-Lapa is more stable in the body and has no toxic side effects compared to when DOX was administered. In addition, by using *β*-lapachone together with a NM prodrug that reacts with H_2_O_2_ and has less toxicity, a synergistic effect and selective expression in cancer cells can be realized [36].

**Figure 7:**
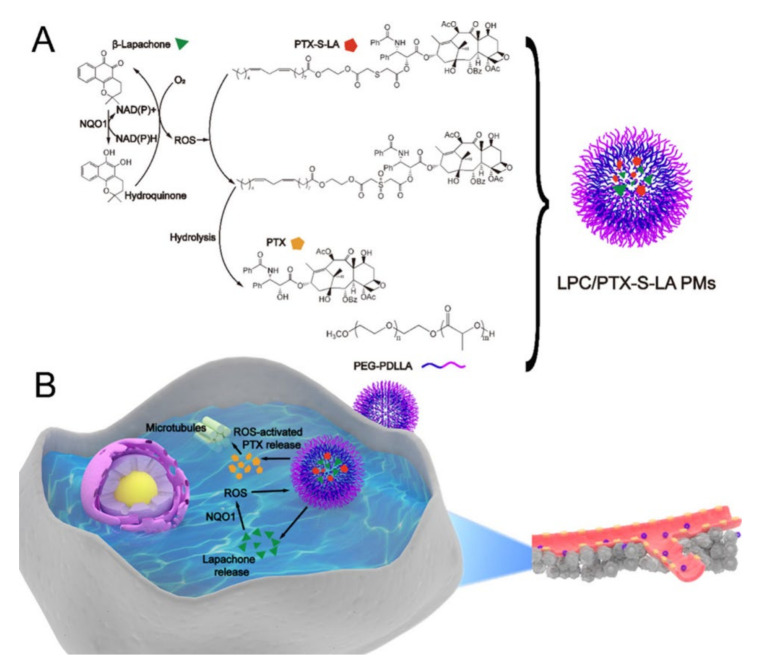
Sequential release by prodrug nanosystem. (**A**) Structure and nanoparticle formation process for *β*-lapachone (LPC), oxidation-responsive thioether-linked linoleic acid paclitaxel conjugates (PTX-S-LA), and PEG-b-poly(d,l-lactic acid) (PEG-PDLLA) (**B**) Upon intravenous injection, LPC/PTX-S-LA polymeric micelles (PMs) migrate into cancer cells due to the enhanced permeability and retention (EPR) effect. These nanoparticles release LPC first, overexpressing nicotinamide adenine dinucleotide (phosphate) (NAD(P)H):quinone oxidoreductase-1 (NQO1), and raising the ROS level. Then, ROS promotes the release of PTX from PTX-S-LA, and PTX induces apoptosis. Reproduced with permission from [37], American Chemical Society, 2019.

In another study by Wang et al., nanoparticles (LPC/PTX-S-LA PMs) with oxidation-responsive thioether-linked linoleic acid PTX conjugates (PTX-S-LA) and *β*-lapachone enclosed in PEG-*b*-poly(d, l-lactic acid) (PEG-PDLLA) as a ROS-responsive drug delivery system were used [37,152,153,154]. Likewise, *β*-lapachone increases ROS, and PTX is released from PTX-S-LA, which effectively acts on cancer cells (Figure 7). When LPC/PTX-S-LA PMs were administered to 4T1 tumor-bearing BALB/c female mice, the tumor growth-inhibitory effect was greater than when taxol was administered. This is because PTX-S-LA showed a high rate of accumulation in tumors by ROS. In addition, no significant change in body weight was observed in mice.

We reviewed a drug delivery system using ROS that can act more effectively against cancer cells than normal cells. The primary feature of sequential drug release using ROS is the selection of drugs in the polymer. Assuming that two drugs are used in combination to form nanoparticles, it is important to select a combination wherein one drug can amplify ROS and the other drug is effectively expressed against the amplified ROS, leading to high anticancer effects. The second feature is the choice of polymer. Above all, it should be able to encapsulate the two drugs of choice, it should be degraded more in cancer cells due to their acidic conditions compared to normal cells, and the polymer should be harmless, biocompatible, and biodegradable.

### 3.2. Sequential Drug Release to Achieve both Intercellular and Intracellular (Spatiotemporal) Drug Release

The aforementioned studies suggested that, even if the drug release profile of NFCDs is ratiometric or sequential, it is difficult to completely treat cancer using these release profiles owing to the characteristics of solid tumor microenvironment (TME). The TME is both heterogeneous and complex as it contains various tumor tissues, including subgroups of genetically diverse cancer cells and nonmalignant stromal cells, which promote tumor cell survival, growth, and resistance to drugs during treatment [155,156,157,158,159]. Therefore, an effective cancer strategy that not only targets cancer cells but also the TME is needed. Thus, taking TME into consideration, NFCDs capable of spatiotemporal controlled release, which is a new paradigm where drugs related to TME treatment are first released in the TME and then internalized into cancer cells to release anticancer agents, are being developed in recent anticancer studies [160].

#### 3.2.1. Spatiotemporal Drugs Release by Matrix Metalloproteinase-2 (MMP-2)

Proinflammatory mediators, such as cyclooxygenase-2 (COX-2) and COX-2-derived prostaglandins 2 (PGE_2_), have a profound effect on tumor survival, growth, metastasis, and angiogenesis [161,162]. Chemotherapy often induces the upregulation of the proinflammatory mediators mentioned above and antiapoptotic genes, causing malignant cells to develop resistance to chemotherapy agents [163,164]. Celecoxib (CXB), a nonsteroidal anti-inflammatory drug can not only affect cancer characteristics by inhibiting the inflammatory COX-2/PGE_2_ pathway, but also reduce chemical resistance by inhibiting antiapoptotic genes [165,166]. Therefore, combination therapy with chemotherapy drugs and nonsteroidal anti-inflammatory drugs can be a new way to change the proinflammatory environment of tumors and to make cancer sensitive to anticancer drugs [167].

Several clinical trials have begun for these combination drugs; it is difficult to achieve a desirable synergistic anticancer effect because anti-inflammatory drugs are generally administered in free form in clinical studies [168,169,170,171]. In addition, the long-term administration of nonsteroidal anti-inflammatory drugs in free form can cause serious side effects [172].

**Figure 8:**
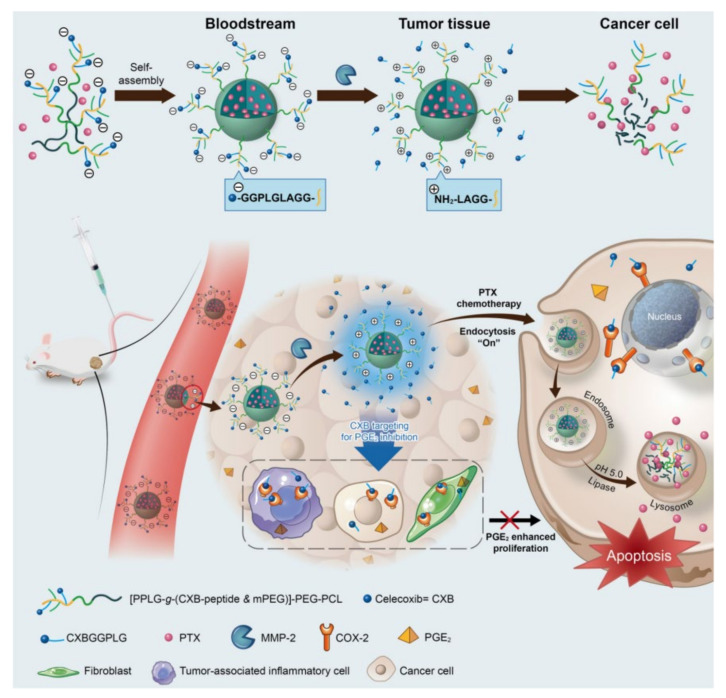
Combinatorial drug-loaded nanospheres capable of spatially sequential drug release using matrix metalloproteinase-2 (MMP-2) sensitive peptide. Celecoxib is first released into the tumor tissue by MMP-2, which is then activated in the tumor environment; paclitaxel (PTX)-loaded nanospheres are positively charged and internalized into cancer cells, and PTX is released intracellularly, causing apoptosis. Reproduced with permission from [38], American Chemical Society, 2019.

To solve these problems, Huang et al. succeeded in achieving spatiotemporal controlled release so that anti-inflammatory agents can act on various cell types and are released from tissues, while chemotherapeutic agents target and are released in cancer cells [38]. Briefly, considering that the peptide with the PLGLAG is sensitive to MMP-2, abundantly present in tumor tissue, CXB was conjugated to the GGPLGLAGG peptide, and the triblock copolymer [PPLG-g-(CXB-peptide & mPEG)]-PEG-PCL (PCxbP) was manufactured through a click reaction with a prepolymer [173,174]. In this triblock copolymer, PTX was encapsulated into the micelle core through the hydrophobic interaction of poly(ε-caprolactone) (PCL) and PTX in an aqueous solution, to complete the MMP-2-sensitive nanospheres loaded with the anti-inflammatory agent CXB and the anticancer agent PTX (MSN-CXB/PTX) [175,176]. After the MSN-CXB/PTX entered the tumor tissue, CXB was released by the concentrated MMP-2, and the negatively charged nanospheres were converted into positively charged nanospheres, which enhanced internalization (Figure 8).

In an in vivo anticancer effect study in nude mice bearing HT-1080 tumor, it was confirmed through tumor size and survival rate that MSN-CXB/PTX showed a better therapeutic effect than the control groups (PBS, MMP-2-insenitive nanospheres loaded with single drug (MIN-PTX, MIN-CXB, and MIN loaded with CXB/PTX (MIN-CXB/PTX)). In addition, quantitative analysis of PGE_2_, COX-2, and anti-apoptotic BCL-2 proteins that induce resistance to chemotherapy in mice tumor tissues showed that MSN-CXB/PTX effectively inhibited the COX-2/PGE_2_ pathway and the expression of BCL-2 proteins, thus having superior tumor cell death compared to control groups [166].

Therefore, MSN-CXB/PTX, which is capable of site-specific release, is considered to be suitable as a cancer cell therapy agent by efficiently solving the resistance problem of cancer cells.

There was another study by He et al. on the spatiotemporal controlled release of NFCDs considering the TME using MMP-2-sensitive peptides [39]. As a combination drug, sunitinib, an angiogenesis inhibitor that has been used with other chemotherapy drugs, is associated with the problem that the target site is different from that of chemotherapy drugs [177,178,179,180,181]. To solve this, the chemotherapy drug PTX was loaded into the core of the pH-sensitive triblock copolymer mPEG-PLLMA(peptide-CD)-PAsp(DBP), and sunitinib was included through *β*-cyclodextrin grafted to the side chain of PLLMA through the MMP-2-sensitive peptide. PTX and sunitinib (ST) co-loaded MMP-2 sensitive peptide micelles (PTX-ST-psMs) capable of site-specific drug release were prepared. When PTX-ST-psMs accumulate at the tumor site, the MMP-2-sensitive peptide is degraded, and ST is released. Then, it moves into cancer cells and sequentially releases PTX, thereby enhancing the antitumor effect. In vitro cell apoptosis and antiangiogenesis studies confirmed that ST can increase the therapeutic effect of PTX on cancer cells. Furthermore, in vivo studies showed that PTX-ST-psMs had superior anticancer efficacy to the control groups (PTX-loaded non-MMP-2 micelles (PTX-Ms), ST-Ms, PTX-ST-Ms), revealing that spatiotemporal controlled release can affect both TME and cancer cells, further increasing the anticancer efficacy.

#### 3.2.2. Spatiotemporal Drugs Release by Dual-pH-Responsive Nanocarriers

Spatiotemporal sequential release can also be achieved by pH control [40]. Dong et al. designed a nanocarrier capable of dual-pH-responsive drug release, by considering the difference between the pH of the tumor vasculature and the pH inside cancer cells. First, DOX is conjugated with polyaspartate (Pasp) through a pH-triggered hydrazone bond to form Pasp-DOX, an inactive macromolecular prodrug, so that DOX can be released in response to the pH conditions inside cancer cells (pH 5–6) [182,183]. A combination of this prodrug and combretastatin A4 (CA4), a tumor vascular inhibitor that can inhibit the growth of tumor vasculature by destroying the tumor cell skeletal structure, was formulated as a pH-sensitive poly(ethylene glycol)-polyhistidine (PEG-Phis) polypeptide. CA4/Pasp-DOX/PEG-Phis is first protonated and then undergoes swelling in response to tumor extracellular pH conditions (pH 6–7) in the tumor vasculature [184]. Protonation and swelling of the formulation cause the release of CA4 from the tumor vasculature to indirectly regulate the TME and promote the uptake of Pasp-DOX/PEG-Phis by tumor cells. Next, after Pasp-DOX/PEG-Phis is internalized by endosomes/lysosomes, the macromolecular prodrug PASP-DOX reacts to the intracellular pH (5.5) condition to release DOX, which then enter the nucleus and causes apoptosis to kill cancer cells. The site-specific drug release of CA4/Pasp-DOX/PEG-Phis was verified by testing its release profile under different pH conditions in PBS and by in vitro noncontact co-culture studies using human umbilical vein endothelial cells and MCF-7/ADR human breast adenocarcinoma cells resistant to DOX. Free DOX and the combination of free DOX/CA4 showed low intracellular accumulation owing to the high expression of P-gp in MCF-7/ADR, whereas CA4/Pasp-DOX/PEG-Phis showed high intracellular accumulation through endocytosis [185].

In a MCF-7/ADR tumor xenograft model, CA4/Pasp-DOX/PEG-Phis showed superior in vivo anticancer efficacy to the controls (free DOX, free CA4, free DOX and CA4 combination, Pasp-DOX, Pasp-DOX + CA4, and CA4/DOX/PEG-Phis). In addition, CA4/Pasp-DOX/PEG-Phis did not show any toxicity against leukocytes. Furthermore, the administration of CA4/Pasp-DOX/PEG-Phis to collagen, a major extracellular matrix component, significantly reduced tumor angiogenesis compared with that of controls, showing a change in the TME [31]. Thus, site-specific sequential drug release from dual-pH-responsive nanocarriers can alter the TME and exert anticancer efficacy against drug-resistant tumors by promoting the accumulation of anticancer agents in the tumor.

In summary, spatiotemporal drug release has the advantage of effectively releasing drugs to the TME and simultaneously releasing anticancer drugs to the cancer cells themselves, enabling effective anticancer treatment. However, there are also limitations in spatiotemporal release studies; only few studies have been conducted and the development has not reached the clinical trial phase.

## 4. Conclusions

Nanoparticles have the capacity to carry therapeutic drugs with various anticancer mechanisms. The anticancer effect of multidrug-loaded nanoparticles varies drastically depending on how drugs are released from nanoparticles. In this review, the pattern of drug release from nanoparticles was investigated and classified in terms of time and space. To this end, we divided drug release patterns into ratiometric drug release, which can deliver drugs to target areas by maintaining a synergistic drug ratio, and sequential release, which releases drugs over time or over space, and we discussed specific physicochemical techniques used to achieve them. Further studies on the release of various drugs from nanoparticles to a suitable location at a suitable time will gradually help increase the anticancer effect of multidrug-loaded nanoparticles and advance the development to the clinical trial phase.

## Figures and Tables

**Table 1:** Characteristics and research progress classified by the release type of nano-formulated combinatorial drugs (NFCDs).

Release Type	Year	Nano Carrier	Used Drug	Research Progress	Author
Ratiometric drug release	2018	VES-g-ε-PLL, dopamine-modified-poly-γ-glutamic acid polymer (γ-PGA-Dopa)	Doxurbicin (DOX), Curcumin	In vitro/in vivo	Xu et al. [28]
	2020	PEGylated ε-poly-l-lysine polymeric nanoparticles	DOX, Lapatinib	In vitro/in vivo	Guo et al. [29]
	2014	Dioleoyl phosphatidic acid, PLGA-PEG-Anisamide NPs	Cisplatin, Gemcitabine monophosphate	In vitro/in vivo	Miao et al. [30]
	2014	Poly(lactic-*co*-glycolic acid) (PLGA) NPs	Rapamycin, Cisplatin	In vitro/in vivo	Guo et al. [31]
	2015	Xyloglucan, tripeptide Gly-Leu-Gly	DOX, Mitomycin C	In vitro/in vivo	Luo et al. [32]
Sequential drug release in intracellular	2017	Hollow mesoporous silica nanoparticles (HMSNs), PEG-PDS-DPA copolymer	Verapamil∙HCl, DOX	In vitro	Palanikumar et al. [33]
	2018	Janus nanoparticles	DOX, Docetaxel	In vitro/in vivo	Zhang et al. [34]
	2017	Poly(ethyleneglycol)-poly [2-(methylacryloyl)ethylnicotinate] (PEG-PMAN)	*β*-Lapachone, ROS-responsive doxorubicin (DOX) prodrug	In vitro/in vivo	Ye et al. [35]
	2019	mPEG-acetalated maltoheptaose (AcMH)Poly(aspartic acid)(PAsp)-AcMH	*β*-Lapachone, Niatrogen mustard (NM) prodrug	In vitro/in vivo	Luan et al. [36]
	2019	PEG-*b*-poly(d,l-lactic acid) (PDLLA)	*β*-Lapachone, Oxidation-resposive thioether-linked linoleic aicd-paclitaxel conjugates (PTX-S-LA)	In vitro/in vivo	Wang et al. [37]
Spatiotemporal sequential drug release	2019	[PPLG-g-(CXB-peptide & mPEG)]-PEG-PCL (PCxbP)	Paclitaxel, Celecoxib	In vitro/in vivo	Huang et al. [38]
	2019	mPEG-PLLMA(peptide-CD)-PAsp(DBP)	Paclitaxel, Sunitinib	In vitro/in vivo	He et al. [39]
	2015	poly(ethylene glycol)-polyhistidine (PEG-Phis) polypeptide	Doxorubicin, Combretastatin A4	In vitro/in vivo	Dong et al. [40]
